# Outcomes of adding second hypoglycemic drug after metformin monotherapy failure among type 2 diabetes in Hungary

**DOI:** 10.1186/1477-7525-6-88

**Published:** 2008-10-31

**Authors:** György Jermendy, the Hungarian RECAP Group, Diana Erdesz, Laszlo Nagy, Don Yin, Hemant Phatak, Sudeep Karve, Samuel Engel, Rajesh Balkrishnan

**Affiliations:** 1 Bajcsy-Zsilinszly Hospital, 3rd Internal Medicine Ward, 1106 Budapest, Maglódi u.89-91, Hungary; 2 Merck Sharpe & Dohme, Budapest, Hungary; 3 Merck & Co., Inc., Whitehouse Station, NJ 08889, USA; 4 The Ohio State University, Columbus, OH 43210, USA; 5 Merck Research Laboratories, Rahway, NJ 07065, USA

## Abstract

**Aim:**

The objective of this observational study was to assess the status of glycemic control and associated patient-reported outcomes in ambulatory Hungarian patients with type 2 diabetes mellitus (T2DM) who were prescribed either a sulfonylurea (SU) or a thiazolidinedione (TZD) in addition to the prior metformin (MF) monotherapy.

**Methods:**

Type 2 diabetics aged ≥ 30 years and who had added an SU or TZD to previous MF monotherapy at least 1 year prior to the visit date were identified during January 2006 to March 2007. Information on HbA1c (A1C), medication use and co-morbid conditions was extracted from the medical record up to 6 months prior to the addition of SU or TZD to MF (baseline), and a minimum of one year after the initiation of either SU or TZD. Glycemic control (A1C < 6.5%) was assessed using the last available A1C value in the medical record. Self-reported hypoglycemia, health-related quality of life (HRQoL) and treatment satisfaction were also assessed.

**Results:**

A total of 414 patients (82% SU+MF and 18% TZD+MF) with a mean age of 60.5 years (SD = 9.4 years) participated in the study. About 27% of patients reported hypoglycemic episodes, with about one-third reporting episodes that resulted into interruption of activities or required medical/non-medical assistance. Three quarters of patients were not at glycemic goal and BMI was the only factor significantly associated with failure to have an A1C level < 6.5%. Patients' HRQoL was significantly associated with self-reported hypoglycemic episodes (p = 0.017), and duration of diabetes (p = 0.045).

**Conclusion:**

Nearly 75% of patients were not at A1C goal of < 6.5% despite using two oral anti-hyperglycemic medications. Approximately 9% of patients reporting hypoglycemia required some kind of medical/non-medical assistance. Greater BMI at baseline was associated with an A1C level ≥ 6.5%. Finally, self- reports of hypoglycemia and duration of diabetes were associated with low HRQoL.

## Introduction

The prevalence of diabetes among adults of age 20 to 79 years was estimated to be 9.7% in Hungary [1,2]. According to the estimate published by the International Diabetes Federation, 11.9% of the Hungarian population will have a diagnosis of diabetes by 2025, making it the country with the highest prevalence of diabetes in Europe [1]. This is worrisome, as those with diabetes have been shown to have an excess risk of mortality compared to those without diabetes [3]. The International Diabetes Federation (IDF) and the European Association for the Study of Diabetes- American Diabetes Association (EASD-ADA) Consensus Algorithm both recommend first line use of metformin (MF) in most patients, with the addition of other drugs to achieve glycemic control if necessary [4-6]. However, one drug is seldom sufficient in the long run, and other pharmacological therapies are often subsequently needed for effective glucose control. Undesirable side effects of antihyperglycemic medications, including hypoglycemia, weight gain and edema, may hinder the ability to achieve or maintain optimal glycemic control [7,8].

As Hungary has been projected to become the European country with the highest prevalence of diabetes by 2025, it would be important to study the status of diabetes management in Hungarian diabetic patients. There is limited evidence, in clinical practice settings, about the effects of various pharmacological treatment options on glycemic control. This is an important issue in metformin-failed patients using thiazolidinedione (TZD), sulfonylurea (SU), or other drugs for glycemic control, as patients treated with those medications may experience hypoglycemia, weight gain, edema or other side-effects.

The objective of this study was to assess the level of glycemic control in clinical practice settings among Hungarian type 2 diabetic patients who were prescribed an SU or TZD after failing to achieve adequate glucose control using MF therapy. We also examined factors associated with inadequate glycemic control in metformin-failed patients. Lastly, we examined factors associated with health-related quality of life, as it was postulated to be adversely affected by side-effects associated with some anti-hyperglycemic medications.

## Materials and methods

### Overview

**Figure 1:**
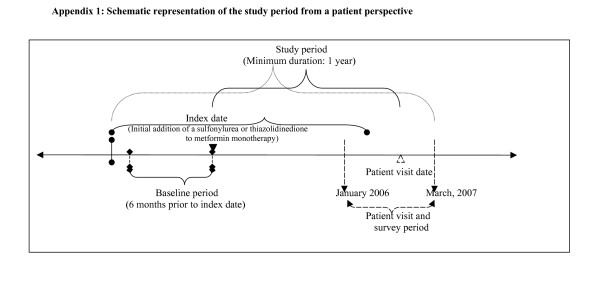
Schematic representation of the study period from a patient perspective.

A schematic representation of the study periods is shown in Figure 1. Type 2 diabetic patients ≥ 30 years of age at the time of type 2 diabetes diagnosis were eligible for participation in this study if they had added either SU or TZD to previous MF monotherapy at least one year prior to the study participation visit that occurred between January 2006 and March 2007. Informed consent was obtained from each patient and study protocol was passed by the human subjects committee at the Bajcsy-Zsilinszly Hospital. Patients completed a survey on the day of their visit to the physician ('visit date'). The date of adding SU or TZD to MF was defined as the 'index date' and the period between the index date and visit date was defined as the 'follow-up period'. The 6-month period prior to the addition of SU or TZD to MF was defined as the 'baseline period'. Information on A1C, medication use and co-morbid conditions was extracted from clinical charts up to 6 months prior to the index date (baseline period) until the current visit date. Based on the IDF (2005) guidelines, an A1C threshold of < 6.5% was used to determine the glycemic control status using the last available A1C value recorded between the index date and visit date ('follow-up period')  [4]. A minimum of at least one year of "follow-up period" was required for each patient. Hypoglycemia and patient quality of life information were assessed based on responses to a patient questionnaire. Patients also evaluated for self-reported of quality of life, treatment satisfaction, and hypoglycemia.

### Subjects

The following inclusion and exclusion criteria were used to select study subjects.

#### InclusioncCriteria

- Diagnosis of type 2 diabetes (ADA criteria [9])

- Age ≥ 30 years at time of type 2 diabetes diagnosis.

- SU or TZD added to MF monotherapy at least one year prior to the visit date

- Patients having required information to complete a minimum core data set**.

- Patients primarily managed in the reporting health care center.

(**) Minimum core data set

1. Patient socio-demographic information: age, gender.

2. Duration of diabetes/age at diagnosis

3. ≥ 1 A1C record within the last year prior to the visit date

4. ≥ 1 A1C record within the Baseline Period (6 months prior to Index Date defined as the date of adding a sulfonylurea or TZD to metformin monotherapy)

5. All glucose-lowering medications (branded and generic names, dosage, dosing frequency, starting and stopping dates) since combination therapy initiation.

#### Exclusion criteria

- Type 1 diabetes.

- Pregnant women/or with gestational diabetes mellitus.

- Diabetes mellitus from generic diseases, surgery, pharmaceutical products, malnutrition, infections and other conditions.

- Insulin therapy at visit date

The following information was collected from retrospective chart review as well as from patient survey which patients filled out at visit date.

#### Glycemic control

glycemic control status was assessed according to the IDF (2005) recommendations of A1C < 6.5% using the last available A1C value during follow-up period.

#### Self-reported hypoglycemia

occurrence of self-reported hypoglycemic episodes in the previous 1 year was determined by patients' responses to a patient questionnaire. Hypoglycemic episodes were categorized as follows:

1. 'Mild': Little or no interruption of activities, and didn't feel the need of assistance to manage symptoms

2. 'Moderate': Some interruption of activities, but didn't feel the need of assistance to manage symptoms

3. The severe symptoms group is a consolidation of the 'severe' and 'very severe' symptoms that were respectively defined as: Felt that you needed assistance of others to manage symptoms (for example, to bring you food or drink), or needed medical attention (for example, called an ambulance, visited an emergency room or hospital, or saw a doctor or nurse).

For evaluating factors associated with self-reported health-related quality of life, patients were also categorized based on self-reporting of hypoglycemia (operationalized as yes/no for this assessment).

#### Self-reported quality of life (EQ5D VAS)

Patient self-reported quality of life information was assessed using the EuroQoL Visual Analog Scale [10].

#### Self-reported treatment satisfaction

treatment satisfaction scores were calculated based on the responses to the "Treatment Satisfaction Questionnaire for Medication" [11].

#### Patient socio-demographic and clinical information

this information was obtained at visit date using a survey instrument. The following variables were collected: age, sex, ethnic origin, height, duration of type 2 diabetes, age at diagnosis, smoking status, alcohol consumption, physical activity, family history, history of macro- and micro-vascular complications and comorbid conditions.

#### Baseline clinical information

Baseline clinical information consisted of following: A1C, fasting plasma glucose, total cholesterol, HDL-C, LDL-C, triglycerides, serum creatinine, urinary albumin excretion rate, systolic and diastolic blood pressure, body mass index, and waist circumference.

#### Previous and current treatment for type 2 DM, switches and reasons for switch

This information was collected during the chart review for the follow-up period and also using a survey filled out by patients at visit date.

#### Co-morbid conditions and information about side-effects

The information on comorbidities was obtained from the medical records. In addition, information about gastrointestinal side-effects, and weight gain was also obtained during the survey.

#### Compliance

Information on patient compliance to the treatment was obtained using the Grant, et al, questionnaire [12].

### Analysis

Descriptive statistics were performed to determine the baseline characteristics of the study population. Appropriate univariate analyses (t-test or χ^2 ^test) were used to compare baseline differences between patients at glycemic goal of <6.5% versus those who were not at glycemic goal during follow up period. Only those factors that exhibited significant association with glycemic goal in the univariate analyses were included in the multivariable logistic regression model. Similarly, univariate and multivariable linear regression analyses were carried out to examine the factors associated with patients' self-reported health-related quality of life. All the statistical analyses were conducted using SAS 9.1 (SAS Institute Inc., Cary, NC, USA) hosted on the Windows platform.

## Results

The baseline characteristics of the patients are described in Table 1. The study cohort consisted of 414 patients with a mean age of 60.5 years (SD = 9.5 years). The mean duration of diabetes was 6.6 years (SD = 4.4 years). The mean A1C at the point of addition of either SU or TZD to MF was 8.2% (SD = 1.5%.) A1C was slightly lower for patients who had TZD added (7.8%, SD= 1.2%) compared to patients who added SU added (8.3%, SD= 1.5%) to metformin. Not surprisingly, the mean A1C levels were lower at the visit date (7.3% ± 1.2%) compared to the index date (Table 2). Approximately 82% of patients were prescribed SU as add-on to MF, and the remaining 18% received TZD. Only 24.2% and 29.0% of MF-failed patients who had SU or TZD, respectively, added to MF were at glycemic goal by the visit date. In this study, 27.6% (n = 114) patients reported hypoglycemic symptoms within the previous 6 months. Among the patients who reported hypoglycemic symptoms, 66.7% (76/114) reported mild hypoglycemic episodes with little or no interruption of activities, 24.6% (28/114) reported moderate hypoglycemic episodes that did interrupt daily activities and 8.7% (10/114) reported severe hypoglycemic episodes that required medical or non-medical assistance.

**Table 1:** Description of Patient Demographic Characteristics at the Index Date When SU or TZD Was Added to Prior Metformin Therapy

**Characteristic**	**All Patients** **N = 414**	SU* + MF^†^ **n = 341**	TZD** + MF^†^ **n = 73**
Age (mean yrs ± std)	60.5 ± 9.5	61.0 ± 8.9	57.9 ± 11.3
Female (%)	49.8%	49.0%	53.4%
Current Smokers (%)	13.5%	13.5%	13.7%
Zero alcohol consumption (%)	42.5%	43.1%	39.7%
Absence of Regular Physical Activity (%)	28.0%	28.7%	24.7%
Physical Activity 3–5 Times/Week (%)	11.6%	12.0%	9.6%
Height (mean cm ± std)	168.0 ± 8.7	168.0 ± 8.6	168.6 ± 9.3
Weight (mean kg ± std)	88.7 ± 15.5	88.5 ± 15.5	90.0 ± 15.4
BMI (mean ± std)	31.2 ± 4.7	31.2 ± 4.7	31.5 ± 4.8
H/O^‡ ^Micro-vascular Complications (%)	8.0%	8.5%	5.5%
H/O^‡ ^Macro-vascular Complications (%)	26.5%	25.8%	27.4%
Years since T2DM Diagnosis (mean yrs ± std)	6.6 ± 4.4	6.7 ± 4.4	6.2 ± 4.3
A1C Level (mean A1C ± std)	8.2 ± 1.5	8.3 ± 1.5	7.8 ± 1.2

* SU: Sulfonylurea

^† ^MF: Metformin

** TZD: Thiazolidinedione

^‡^H/O: history of

**Table 2:** Description of Patient Clinical Characteristics at the Visit Date

**Characteristic**	**All Patients** **N = 414**	SU* + MF^†^ **n = 341**	TZD** + MF^†^ **n = 73**
A1c at on the follow-up period (mean ± std)	7.3 ± 1.2	7.4 ± 1.3	7.0 ± 1.2
Patients at A1C Goal (%)	24.88%	23.75%	30.14%

**Therapy patients were using at the time of visit (also referred as "current therapy") – (%)**			

Monotherapy (%)	3.16%	3.24%	2.78%
*Combination Therapy*			
Metformin + Sulfonylurea (%)	57.52%	69.12%	2.78%
Metformin + TZDs (%)	16.26%	3.24%	77.78%
Sulfonylurea + TZD (%)	0.24%	0.29%	0.00%
Sulfonylurea + Alpha glucosidase inhibitors (%)	0.73%	0.88%	0.00%
Sulfonylureas + TZD + Metformin (%)	11.89%	12.65%	8.33%
Sulfonylureas + Alpha glucosidase inhibitors + Metformin (%)	5.83%	7.06%	0.00%
Metformin + TZD + Alpha glucosidase inhibitors (%)	1.46%	0.59%	5.56%
Sulfonylureas + TZD + Metformin + Alpha glucosidase inhibitors (%)	2.91%	2.94%	2.78%

Note: These patients received SU or TZD after failing metformin at index date. Current therapy can be different than this combination, as patients may have received other therapies in addition to SU or TZD. For the purpose of this study, each patient was required to have a minimum of 1 year of time-period between the index date and the visit date. Patients receiving insulin during that period were excluded from this study.

* SU: Sulfonylurea

^† ^MF: Metformin

** TZD: Thiazolidinedione

### Glycemic control

Three quarters of patients with T2DM were not at glycemic goal at the visit date. Table 3 describes the association between glycemic goal status and patient reported outcomes. Patients not at A1C goal were less likely to report taking medication exactly as prescribed (p = 0.043) compared to patients at goal. Patients not at A1C goal were also more likely to be bothered by medication side-effects (p = 0.024). Patients not at goal were also less likely to be satisfied with effectiveness of therapy (p = 0.003) and reported lower global satisfaction score (p = 0.012) than patients at goal. Multivariate logistic regression models results are shown in Table 4. Patients not at glycemic goal were more likely to have a higher BMI at baseline as compared to patients at glycemic goal (p = 0.009).

**Table 3:** Association of At-Goal A1C and Patient Reported Outcomes

**Characteristic**	**N**	**Patients At Goal**	**Patients Not At Goal**	**p-value^&^**
Self-reported hypoglycemic episodes				

Patients with Hypoglycemic Symptoms (%)	114	27.5%	27.7%	0.968
Patients without Hypoglycemic Symptoms (%)	299	72.5%	72.3%	

Symptom Severity				

None (%)	299	72.5%	72.4%	0.663
†Mild resulting into no or little interruption in activities (%)	76	19.7%	18.0%	
†Moderate resulting into interruption in daily activities (%)	28	7.8%	6.4%	
*†Severe requiring some kind of medical or non-medical assistance (%)	10	0%	3.2%	

EQ VAS Score (mean ± std)	414	77.0 ± 16.5	76.7 ± 15.5	0.854

Adherence & Barriers to Adherence				

Always taking EXACTLY as prescribed (%)	221	62.1%	50.7%	0.049
Never UNSURE about instructions (%)	323	80.6%	77.7%	0.534
Never UNABLE to follow plans (%)	313	82.5%	73.8%	0.072
Never BOTHERED by side effects (%)	266	73.8%	61.5%	0.024
Never PROBLEMS getting Rx filled (%)	379	95.2%	91.5%	0.230

Satisfaction with Treatment				

Effectiveness (mean ± std)	406	71.1 ± 16.3	65.9 ± 15.0	0.003
Side Effects (mean ± std)	410	92.7 ± 14.9	90.1 ± 17.0	0.174
Convenience (mean ± std)	412	67.3 ± 20.6	67.3 ± 17.8	0.986
Global Satisfaction (mean ± std)	113	75.5 ± 15.4	70.8 ± 16.3	0.012

% are based on column.

^‡ ^Based on the Chi-square test of the null hypothesis of no association between patient reported experience of hypoglycemia and treatment adherence and barriers to adherence.

^&^Based on the Wald test of the null joint hypothesis of no association of the severity symptoms with adequate glycemic control (i.e. all coefficients are equal to zero).

^¶ ^Based on the t-test of the null hypothesis of no association between patient reported experience of hypoglycemia and specified characteristics * Reference category

^† ^{Mild: Little or no interruption of activities, and didn't feel the need of assistance to manage symptoms, Moderate: Some interruption of activities, but didn't feel the need of assistance to manage symptoms, Severe: The severe symptoms group is a consolidation of the 'severe' and 'very severe' symptoms that were respectively defined as: Felt that you needed assistance of others to manage symptoms (for example, to bring you food or drink), and needed medical attention (for example, called an ambulance, visited an emergency room or hospital, or saw a doctor or nurse)}.

**Table 4:** Factors associated with glycemic goal – logistic regression analyses

**Variable**	**Odds ratio**	**95% Wald confidence limits**		**Pr > ChiSq**
BMI at baseline^‡^	0.92	0.86	0.98	0.009
H/O Macro-vascular complications (Yes)^¥^	1.45	0.82	2.57	0.205
Females^¥^	0.72	0.41	1.29	0.272
Absence of Regular Physical Activity^¥^	0.77	0.41	1.47	0.433
Zero alcohol consumption^¥^	0.93	0.51	1.71	0.815
Zero cigarette consumption^¥^	1.14	0.64	2.04	0.649
Number of Co-medications	0.87	0.58	1.29	0.483
MF + TZD at index date^¥^	1.53	0.81	2.89	0.195
Family history of diabetes^¥^	0.70	0.41	1.22	0.209

(Probability modeled is Goal = Yes. N = 304)				

^† ^MF: Metformin

** TZD: Thiazolidinedione

^‡ ^Body Mass Index

^¥ ^(Reference categories: absence of macro event, male, some physical activity, occasionally/daily drinker, current/past smoker, Metformin + Sulfonylurea at baseline, no family history of diabetes)

### Self-reported health-related quality of life

Self-reported health-related quality of life (EQ-5D VAS score) was similar for patients at goal (77.0 ± 16.5) and for those not at goal (76.7 ± 15.5, p = 0.854) (Table 3). When factors affecting patients' health-related quality of life were assessed, it was found to be negatively associated with patients' reporting of hypoglycemia (yes/no) (p = 0.017) and duration of diabetes (p = 0.045) (Table 5).

**Table 5:** Factors associated with patient quality of life (EQ5D VAS) – linear regression analyses

**Variable**	**Coefficient**	**p-value**
Age	-0.10	0.318
Hypoglycemic episodes (Yes)	-4.66	0.017
H/O Macro-vascular complications	-2.18	0.257
Years since T2DM Diagnosis	-0.41	0.045
Metformin+TZD at index date	4.81	0.046

N = 346		

The adjusted linear regression reported in model contains all variables that were significant at p ≤ 0.20 in the univariate analysis. Instead of the hypoglycemia symptom severity effects it contains an indicator for hypoglycemia

## Discussion

This is the first study to evaluate glycemic control in metformin-failed patients in clinical practice in Hungary. Approximately 75% of patients were not at glycemic goal after the addition of sulfonylurea or thiazolidinedione to their metformin monotherapy. Similar findings were reported in a study by Cook et al which evaluated the impact of combination therapy (metformin and sulfonylurea) on glycemic control [13]. Glycemic control has been shown to continue to deteriorate 6 months after the addition of sulfonylurea to the metformin monotherapy [13]. This is of great concern as it exposes patient to the increased risk of hyperglycemia related complications. In addition, we found that patients not at glycemic goal reported being less likely to take medications exactly as prescribed, and to have lower global treatment satisfaction scores. This is in line with findings of other studies [14,15]. In this study, patients not at glycemic goal were more likely to report higher BMI at baseline. It is possible that patients with higher BMI did not optimally use anti-hyperglycemic treatments, including SU or TZD, as these treatments are often associated with further weight gain [16,17].

Patients not at A1C goal were more likely to be bothered by side-effects as compared to patients at A1C goal. Another important aspect that may be affected by side-effects is patients' self-reported health-related quality of life. In this study, we found a negative association between reporting of hypoglycemia and self-reported health-related quality of life (p = 0.02). Patients reporting hypoglycemia were more likely to report experiencing side-effects including weight gain, excessive fatigue, dizziness, shakiness and abdominal pain (data not shown). All these factors could have contributed to patients with reported hypoglycemia having lower quality of life than patients who did not report hypoglycemia. Our findings are similar to other studies that have shown an association between reports of hypoglycemia and reduced quality of life [18-20].

Finally, oral anti-hyperglycemic drugs without hypoglycemia or weight gain may also help patients with type 2 diabetes in achieving the glycemic target of <6.5%. Inadequate control of glucose levels has been associated with development of complications in diabetes patients. Studies have found that improved glycemic control benefits people with both type 1 or type 2 diabetes. The United Kingdom Prospective Diabetes Study (UKPDS) findings suggest that every percentage point drop in glycosylated hemoglobin (A1C) blood test results (e.g., from 8.0% to 7.0%) was associated with a reduction in risk of micro-vascular complications by 37%, myocardial infarction by 14%, and heart failure by 16% [21]. Therefore it would be important that patients failing metformin therapy receive additional antihyperglycemic agents to reduce A1C and minimize the risk of cardiovascular events. Augmentation of anti-hyperglycemic therapy in metformin-failed patients using sulfonylurea or TZD is often associated with either increase in the body weight and/or hypoglycemia [22-25]. Based on the estimates published by the American Diabetic Association, in the years 2001–2003, 57% of patients diagnosed with diabetes were treated with oral anti-hyperglycemic medications [26]. This propensity of physicians to use oral anti-hyperglycemic agents warrant the use of effective drugs, preferably without undesirable side-effects including weight gain or hypoglycemia.

Certain study limitations deserve note. Our study was an observational study, and even though detailed confounder adjustment was made, we cannot infer causality from our study findings. Also, because of the limitations of the study protocol and to limit administrative burden of the patient survey, we were not able to collect detailed information on factors such as explicit reasons for patient medication changes. In spite of these minor limitations, the findings of this study have important implications for treatment of patients with diabetes.

## Conclusion

In conclusion, this observational study of diabetic patients in Hungary found that 3 out of 4 patients were not at glycemic goal (A1C <6.5%) despite using combinations of oral anti-hyperglycemic medications. Patients not at glycemic goal were more likely to report side effects related to their medication. Severe hypoglycemia requiring some kind of medical or non-medical assistance was reported by approximately 9% patients reporting hypoglycemic episodes. Patients reporting hypoglycemia were also more likely to report lower levels of satisfaction with medication side-effect profiles as well as lower health-related quality of life.

## Authors' contribution

GJ: conceptualization, design, data collection, manuscript revision. DE: conceptualization, design,  manuscript revision. LN: conceptualization, design,  manuscript revision. DY: design, data analysis. HP: conceptualization, design,  manuscript revision. SK: data analysis and manuscript writing. SE: conceptualization, design,  manuscript revision. RB: design, data analaysis, manuscript writing, revision.

## Competing interests

The authors declare that they have no competing interests.
